# Development of micro-propagation and mini cutting protocol for fast growing Melia, Dalbergia and *Eucalyptus* clones for pulpwood and bio-energy plantations

**DOI:** 10.1186/1753-6561-5-S7-P131

**Published:** 2011-09-13

**Authors:** Senniappan Chinnaraj, C Malimuthu

**Affiliations:** 1 S.V. Subrahmanyam Tamil Nadu Newsprint and Papers Ltd, India

## 

Per capita paper consumption is only 9.2 kg in India, which is much lower when compared to other neighboring developing economies, such as, China (42 kg) & Indonesia (23 kg). Currently Indian economy is growing by 7.5% per annum. Along with economical growth, the paper demand is also expected to grow and cross 20 million tonnes per annum by 2020 from current 10 million tonnes. Availability & quality of raw material, non availability of land for pulp wood plantations, alternate use of pulp wood for other uses like bio-energy and regulations to increase green energy use in mill operations would become major challenge for Indian paper industry’s survival and growth. To face these changes effectively, paper industries in India initiated many social forestry models and brought 4,00,000 hectors under pulp wood plantations. This has helped pulp wood availability and reduced the fibrous raw material shortage to some level but it is not enough to meet the growth.

Tamil Nadu Newsprint and Paper Limited (TNPL) is state owned integrated pulp and paper industry situated in southern most state of India and produce around 4,00,000 tons of paper per annum. TNPL has 300 Tonnes per day (TPD) hardwood fibreline along with 550 TPD bagasse fibre line to meet its pulp requirement. TNPL had setup biotechnology and bio-energy research facility under the existing R & D Department to work on identification, selection and multiplication of improved pulpwood clones of *Eucalyptus* and other alternate fast growing hardwood species through modern biotechnological methods to meet pulpwood and also bio-energy need. The plant tissue culture facility has one million plants per annum capacity and optimized protocols for many improved *Eucalyptus* pulp wood clones to use as clonal mother plants at Clonal Production and Research Centre of Plantation Department using mini cutting process. TNPL clonal production facility has capacity of 15 million seedlings per annum and already covered 52,000 acres of pulp wood plantation and plan to cover additional 15,000 acres every year.

As a part ongoing research activity, one *Melia dubia* clone for bio-energy/pulpwood and one *Dalbergia sissoo* clone and three clones of eucalyptus, (each one from *E. urograndis*, *E. tereticornis*, and *E. camaldulensis*), were identified based on its biomass productivity, as well as, pulp quality and yield. Micro-propagation protocol was optimized for all the above five clones with modified MS medium with various concentrations of cytokinin and auxin. The seedlings produced using micro-propagation were used as mother plants for clonal mini garden in the concrete sand beds where the nutrient requirements where optimized for further multiplication using mini cutting process. The results of above study is presented and discussed in this paper.

Wood and pulp properties of all selected *Melia dubia*, *Dalbergia sissoo*, *E. urograndis*, *E. tereticornis*, and *E. camaldulensis* clones are presented in the Table 1 along with reference *Eucalyptus* pulp wood used in the mill. All the selected clones show high yield and strength properties. However, *Melia dubia* is not preferred as pulpwood due to low bulk density which would result in less through-put in fibreline. But it can be exploited for bio-energy applications especially for biomass gasification to generate producer gas and use in Lime Kiln to replace fuel oil and also for other wood product applications. All the selected clones give approximately minimum 2.5% more pulp yield with improved pulp properties when compared reference pulpwood currently used in the mill. Higher pulp yield would definitely give better economy and environmental performance of mill operations. For example, 1 % yield increase would result in annual savings of INR 35.0 million in pulp wood cost for mill of our size i.e. 300 TPD. Therefore, for 2.5 % yield increase would result in annual saving of around INR 87.5 million apart from chemical saving and other environmental benefits.

**Table 1:** Results of wood pulping and pulp properties of selected pulpwood and bio-energy clones

Parameters	Units	***Melia dubia***	***Dalbergia sissoo***	***Eucalyptus urograndis***	***Eucalyptus tereticornis***	***Eucalyptus camaldulensis***	***Reference***
Bulk density	kg/m3	136	230	184	213	220	225
Basic density	kg/m3	318	547	402	502	520	510
Chemical addition	%	15.0	15.0	15.0	15.0	15.0	15.0
Screen rejects	%	0.8	0.7	0.2	0.2	0.8	0.8
Screened pulp yield	%	46.5	47.0	46.6	47.3	47.5	44.2
Kappa Number		21.6	18.5	20.4	19.1	19.7	25.3
Brightness	% ISO	27.8	30.7	29.1	34.9	30.5	24.2
Unbleached Strength properties at 300ml CSF							
Tensile index	Nm/g	94.2	79.0	98.0	92.0	95.0	74.0
Tear index	mN.m2/g	11.9	9.3	8.7	10.3	9.5	8.2
Burst index	kPa.m2/g	6.4	5.6	6.9	6.5	5.6	5.1

To take all the selected clones from lab to land in a short period, micro-propagation and mini cutting protocols were optimized. The results are presented in the Table 2. *E.tereticornis* clone found to perform well during initiation, elongation and hardening*.*On the other hand , *E. urograndis* found to perform well during multiplication and gave maximum number of shoots per clump. All the *Eucalyptus* clones found to perform well during the rooting. *Melia dubia* and *Dalbergia sissoo* are found to perform poor in micro-propagation compared to *Eucalyptus.* Seedlings produced by micro-propagation are used for mini cutting experiments and results are presented in Table 2. *E. urograndis* produced more number of cuttings per plant per month followed by*Dalbergia sissoo*, *E.tereticornis E. camaldulensis* and *Melia dubia.* Rooting and survival rate is also high for *Eucalyptus* clones when compared to*Dalbergia sissoo* and*Melia dubia.*

**Table 2:** Results of micro-propagation and mini cutting studies of selected pulpwood and bio-energy clones

Parameters	Units	***Melia dubia***	***Dalbergia sissoo***	***Eucalyptus urograndis***	***Eucalyptus tereticornis***	***Eucalyptus camaldulensis***
**Imitation**						
Optimum Benzylaminopurine	mg/lit.	1.0	1.0	1.5	0.5	0.5
Initiation rate	%	56	36	48	84	64
Average shoot length after 30 days	cm	1.0	1.5	1.0	3.0	1.5
**Multiplication**						
Optimum BAP	mg/lit.	0.25	0.15	0.15	0.15	0.15
Average Number of shoots per clump	no	2.0	3.0	32	24	15
Average shoot length	cm	3.0	2.5	0.5	1.5	1.0
**Elongation**						
Optimum Auxin	mg/lit.	0.0	0.0	0.0	0.5 (IAA)	4.0 (NAA)
Average Number of shoots per clump	no	2.0	2.0	4.0	6.0	4.0
Average shoot length	cm	5.0	5.0	3.0	7.0	4.0
**Rooting**						
Optimum IBA	mg/lit.	1.0	1.0	1.0	1.0	1.0
Average Number of roots per shoot	no	4.0	4.0	8.0	7.0	5.0
Average shoot length	cm	2.0	1.5	2.0	5.0	3.0
Rooting	%	90	40	100	100	100
**Hardening**						
Survival	%	62	45	85	95	80
**Mini cutting**						
Number of shoots per plant/cutting	no	4.0	7.0	8.0	6.0	5.0
Rooting and survival	%	70	63	79	86	73

Micro-propagation followed mini cutting protocol for propagation of all the above clones has been successfully adopted by mill for commercial production of quality seedling for mill’s pulp wood and other plantation programme.

